# Sorting cells by their dynamical properties

**DOI:** 10.1038/srep34375

**Published:** 2016-10-06

**Authors:** Ewan Henry, Stefan H. Holm, Zunmin Zhang, Jason P. Beech, Jonas O. Tegenfeldt, Dmitry A. Fedosov, Gerhard Gompper

**Affiliations:** 1 Theoretical Soft Matter and Biophysics, Institute of Complex Systems and Institute for Advanced Simulation, Forschungszentrum Jülich, 52425 Jülich, Germany; 2 Division of Solid State Physics, NanoLund, Lund University, PO Box 118, S-221 00 Lund, Sweden

## Abstract

Recent advances in cell sorting aim at the development of novel methods that are sensitive to various mechanical properties of cells. Microfluidic technologies have a great potential for cell sorting; however, the design of many micro-devices is based on theories developed for rigid spherical particles with size as a separation parameter. Clearly, most bioparticles are non-spherical and deformable and therefore exhibit a much more intricate behavior in fluid flow than rigid spheres. Here, we demonstrate the use of cells’ mechanical and dynamical properties as biomarkers for separation by employing a combination of mesoscale hydrodynamic simulations and microfluidic experiments. The dynamic behavior of red blood cells (RBCs) within deterministic lateral displacement (DLD) devices is investigated for different device geometries and viscosity contrasts between the intra-cellular fluid and suspending medium. We find that the viscosity contrast and associated cell dynamics clearly determine the RBC trajectory through a DLD device. Simulation results compare well to experiments and provide new insights into the physical mechanisms which govern the sorting of non-spherical and deformable cells in DLD devices. Finally, we discuss the implications of cell dynamics for sorting schemes based on properties other than cell size, such as mechanics and morphology.

The ability to sort specific cells from heterogeneous populations of bioparticles is highly coveted in biomedical fields such as diagnostics and cell biology. Many of the standard cell sorting techniques, such as fluorescence- and magnetic-activated cell sorting devices^1^,^2^, are labor intensive, use cumbersome and expensive equipment, and require preliminary cell-labeling stages. In contrast, lab-on-a-chip technologies operate at a micrometer scale and provide a promising alternative to the current cell-sorting approaches. Microfluidic devices have multiple advantages over conventional approaches including reduced manufacturing costs, a smaller sample volume requirement, and the ability to sort cells based on their intrinsic properties, leading to an increased automation of the sorting process.

One increasingly popular microfluidic technique, pioneered by Huang *et al*.^3^, is called deterministic lateral displacement (DLD) and utilizes micropost arrays to continuously sort particles according to their size^4^,^5^,^6^,^7^. Various geometries of DLD arrays can be characterized by a critical separation radius *R*_*c*_^8^, yielding a highly reliable method for separating rigid spherical particles of different sizes. Thus, sorting in DLDs arises as particles smaller than *R*_*c*_ are able to travel with the flow and swap between pillar lanes (i.e., lane swapping, where a lane is defined as a straight path running parallel alongside a row of pillars) in a zig-zag motion with nearly zero lateral displacement (neutral zig-zag mode, Fig. 1(a)), while particles larger than *R*_*c*_ are displaced laterally with respect to a driving fluid flow (displacement mode, particles remain in one lane without swapping, Fig. 1(a))^3^. The critical radius can be inferred from the streamlines of the fluid flow without the presence of particles (as illustrated in Fig. 1(b)) and has also been determined empirically^8^. Furthermore, different DLD devices have already been used successfully to separate biological particles and cells. For example, separation of red blood cells (RBCs), white blood cells and platelets from whole blood has been demonstrated^4^, and parasitic trypanosomatids have been extracted from blood samples^5^.

In the above examples, the considered sorting parameter is effective size. However, many bioparticles are non-spherical and deformable, and the idea of a single effective particle radius depends on the particle’s orientation and the degree of deformation experienced in flow^9^,^10^,^11^. Furthermore, biological particles may undergo some type of periodic dynamical motion in shear flow (e.g., tumbling) with a constantly changing effective radius, which makes the prediction of sorting on the basis of a particle-free flow not possible. The potential variability of the effective size is illustrated in Fig. 1(c) for RBCs which have a rich dynamic behavior and deformability in a pillar array, and it is clear that the sorting of other such bioparticles will be similarly complex. Therefore, the effects of cell dynamics must be carefully considered in microfluidic devices and can even be used for novel sorting schemes which are sensitive to the mechanical properties of cells. Investigations on white blood cells in DLD devices have already indicated that deformations have an impact on their transit behavior^12^. Furthermore, RBC deformability has been suggested as a sorting parameter^13^ and recent numerical simulations^14^,^15^,^16^ of elasticity-based sorting have considered this possibility. Targeting these elastic, structural, and dynamical characteristics for sorting adds a new level of complexity to designing DLD devices and testing the myriad of combinations between cell types and device configurations experimentally would be expensive and impractical. Consequently, there is a compelling need for theoretical methods and simulations to understand the essential physical mechanisms and to make quantitative predictions of how cell dynamics will affect transit behavior of bioparticles in DLD devices.

In this work, we perform a combined numerical and experimental investigation of RBC sorting in DLD devices. Instead of analyzing the location of RBCs after they exit the pillar array, as is customary with DLD devices, we focus on quantitatively describing the entire trajectory of RBC transit through devices. We find that a description of trajectories through the two modes (i.e., displacement and neutral zig-zag, shown in Fig. 1(a)) is far too simple to adequately describe sorting of deformable and anisotropic particles. Even though the displacement mode defined by device geometry remains similar to previous experiments with rigid spherical particles, we observe a range of zig-zag modes in experiments and simulations, which can lead to positive, neutral and negative displacements of a RBC within different configurations of the device. Furthermore, the examination of RBC dynamic behavior immediately before and after lane-swapping events in DLDs allows us to identify a relationship between RBC trajectory, its dynamic behavior and hydrodynamic interactions. This relationship is further investigated by varying the ratio between intracellular and extracellular viscosities, as viscosity contrast is known to dramatically affect RBC dynamics^17^. Indeed, by invoking a different dynamic behavior such as tumbling or tank-treading, we show that it is possible to rationalize and control RBC trajectories in DLD devices. Thereby, we can propose a novel sorting strategy and show how a reliable simulation method for deformable and anisotropic particles through DLD devices becomes a powerful tool for designing sorting-schemes which depend on particles’ dynamic behavior and hence, their intrinsic mechanical characteristics, such as rigidity and internal viscosity.

## Results

**Figure 1:**
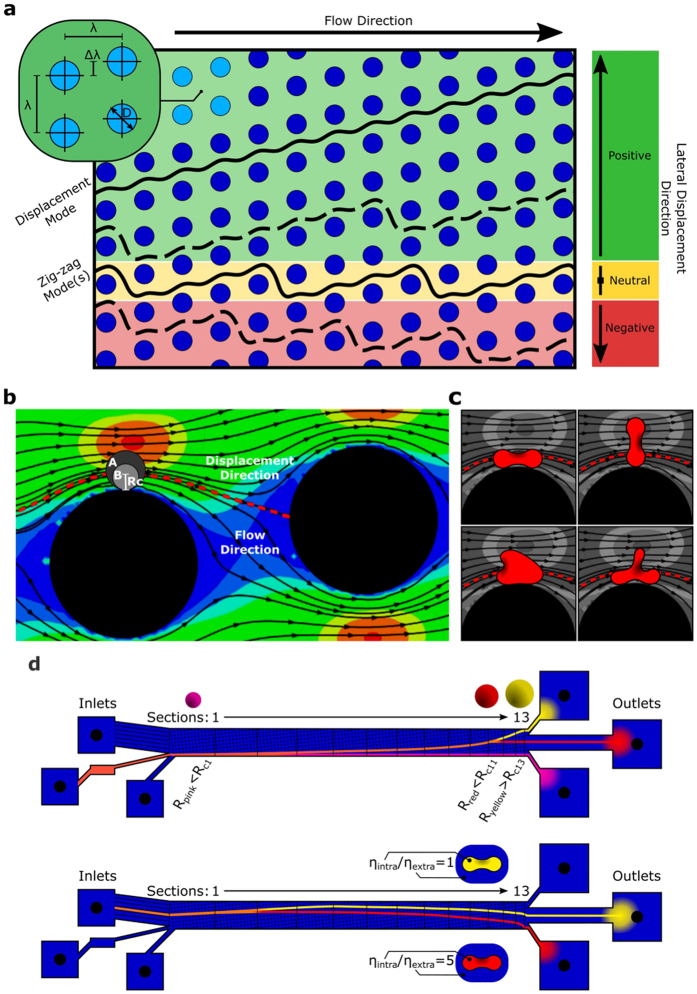
DLD sorting of rigid spheres vs RBCs. (**a**) The possible trajectories of particles traversing an obstacle array defined by the central post-to-post distance *λ*, row shift Δ*λ*, and post diameter D. Solid lines represent the displacement (no lane swapping) and neutral zig-zag (swapping between lanes, where a lane is defined as a straight path running parallel alongside a row of pillars) modes available to rigid spherical particles. Anisotropic deformable particles have access to many additional zig-zag modes which allow for positive or negative lateral displacement, two of which are shown as dashed lines. **(b)** The flow field of a fluid driven from left to right, past two pillars of a DLD device obstacle array. The contour colors from blue to red correspond to the strength of fluid velocity in the flow direction. The red dotted line shows the separatrix between flow traveling over or under the second pillar. Rigid spherical particle A, with 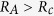, is carried over the pillar, in a displacement mode. Particle B, with 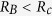, is carried under the pillar assuming a neutral zig-zag mode^3^,^8^. **(c)** Deformable and anisotropic RBCs can flow above or under the separatrix depending on their orientation and deformation; dynamic characteristics which change as they interact with the flow. **(d)** Schematic behavior of particles in a DLD device with 13 successive sections, each with larger *R*_*c*_: when the device is used to sort 3 sizes of rigid spherical beads, each size undergoes a transition from the displacement mode to a neutral zig-zag mode in a different section. The orange color corresponds to an initially polydisperse suspension of different spheres, while the other colors depict separated monodisperse fractions at the end of the device. The same device sees very different separation trajectories when sorting RBCs undergoing different types of dynamic behavior. Dynamic behavior can be controlled by changing the viscosity contrast between interior and extracellular fluids. Note that due to the presence of negative zig-zag modes for RBCs it is necessary to use the large central inlet to accommodate separation in both directions.

The experimental and simulation results are presented in unison to facilitate a detailed comparison. Initially the possible transit modes available to RBCs in two different DLD devices are considered, as these modes are different from the displacement and neutral zig-zag modes available to rigid spheres. The first device is thick enough to allow RBCs to explore a full range of dynamic behavior, while the second is sufficiently thin to force horizontal orientation of RBCs, suppressing reorientation dynamics due to confinement. A schematic of the device structure and pillar array geometry can be seen in Fig. 1 and Supplementary Figure S1. Both devices have 13 sequential sections, each housing a pillar array defined by the post diameter *D* = 20 μm, central post-to-post distance *λ* = 32 μm, and row shift Δ*λ*, which increases from a value of 0.8 μm to 8.8 μm incrementally between subsequent sections (see Supplementary Table S1). We explore how the transition between different transit modes occurs at different sections in the device depending on RBC dynamic behavior, which is controlled directly using the different confinements of the thin and thick devices, and by changing the ratio *C* = *η*_*i*_/*η*_*o*_ between intra-cellular viscosity of the RBCs and extra-cellular medium. Finally, we discuss qualitatively why the different dynamic behavior arises and how it results in different transit behavior.

The transit of deformable anisotropic particles through the 13-section device is considerably different from that of rigid spheres, as depicted schematically in Fig. 1(d). This is not only due to transitions from the displacement to zig-zag modes occurring in different sections but also due to the availability of additional zig-zag modes. Ultimately, there is no reason why a particle should prefer the neutral, zero lateral-displacement, zig-zag mode; depending on the frequency of lane swapping, negative, neutral, and positive net lateral displacement can be induced.

### DLD transit modes in the thick device

**Figure 2:**
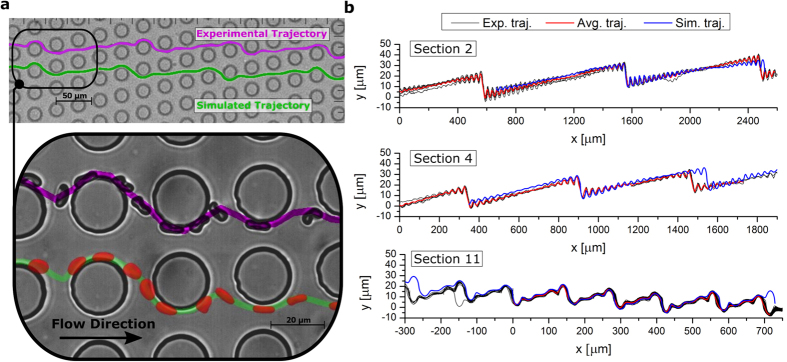
Experimental and simulated trajectories for RBCs at viscosity contrast C = 5. **(a)** The trajectories of the experimental and simulated RBCs are obtained by following their center of mass through the pillar array. **(b)** Experimental and simulated RBC trajectories for several sections of the thick device. Black lines show multiple aligned experimental RBC trajectories and red lines correspond to average experimental trajectories. The blue lines show the RBC trajectories found in simulations. Section 2 shows an approximately neutral zig-zag mode, section 4 illustrates a positive zig-zag mode, and section 11 leads to a negative zig-zag mode.

In order to understand the nature of the transit modes available to RBCs, we follow the trajectory of a RBC’s center of mass through the obstacle array, by recording the *x* and *y* coordinates, along and perpendicular to the flow direction, respectively. Figure 2 depicts a selection of different zig-zag modes of RBCs in different sections of the thick DLD device at a physiological viscosity contrast of *C* = 5. Figure 2(a) shows a side by side comparison of simulated and experimental RBC snapshots and a zig-zag trajectory in section 11, which is defined by a row-shift of Δ*λ* = 6.8 μm. The high frequency of lane-swapping in the RBC trajectory makes it a good introductory example of a zig-zag mode because it is possible to view it with isometric scaling. This type of data representation will form the basis for comparisons between experimental and simulated results. In other sections of the devices, trajectories might have periodicity at length scales too large for isometric visualization. Consequently, the trajectories seen in Fig. 2(b) are displayed with different *x* and *y* scaling. This allows us to present a complete, albeit distorted, depiction of the trajectories. Furthermore, the experimental trajectories in Fig. 2(b) have been selected from a population of recorded trajectories in accordance with pre-screening criteria, which were used to remove trajectories of insufficient length as well as trajectories where inter-RBC interactions were detected. Further details about the data sample and the pre-screening criteria can be found in the Supplementary Information.

Figure 2(b) compares experimental and simulated RBC trajectories in various sections of the thick DLD device and nicely presents the range of different zig-zag modes which we observe. In order to quantitatively describe and differentiate these zig-zag modes, we define their average lateral displacement, *l*, per post encounter. The value of *l* can be calculated by considering the competing effects of lane-swapping events and lateral displacement induced by the geometry of the pillar array. The positive lateral displacement per post is simply Δ*λ* and the average displacement per post encounter due to lane-swapping is given by the frequency of lane-swapping events multiplied by the distance between rows, −*λf*_*i*_. The additive effect of these two motions yields


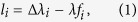


the average lateral displacement per post encounter in the *i*^*th*^ section of the device. The frequency *f*_*i*_ of lane-swapping is determined by the inverse of the average number *m* of post encounters per lane-swapping event. Theoretically, *m* could have any positive value, where the special cases *m* = ∞ and m = *λ*/Δ*λ* yield the ideal displacement and neutral zig-zag modes discussed previously.

Using the calculation for *l*_*i*_ shown in Eq. (1), we return to Fig. 2 and quantitatively describe the observed zig-zag modes; in section 2 and 4 with Δ*λ*_*i*_ = 1.2 μm and Δ*λ*_*i*_ = 2.0 μm, we find nearly neutral zig-zag modes with values of 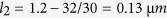 and *l*_4_ = 0.22 μm, close to the ideal case; finally section 11 induces a negative zig-zag mode, where lateral displacement is offset by the lane-swapping events and the average displacement per post is *l*_11_ = 6.8 − 32/4.4 = −0.47 μ*m*. Note that *m* takes a non-integer value in this case, because the periodicity between lane-swapping events is irregular. The irregular periodicity supports the idea that changes in RBC orientation and deformation cause it to explore more than one flow stream and exhibit more complex behavior than simple hard spheres. However, it is important to emphasize that the non-integer *m* values we find are not the result of random RBC behavior in the device. The behavior of RBCs is governed by local fluid flow and their elastic and viscous properties, which is supported by RBC dynamics in shear^18^,^19^ and tube^17^ flows, and therefore, there is no reason to expect random RBC dynamics in DLDs. The only randomness arises from the initial orientation and position of the RBCs as they enter the device, and the thermal fluctuations which result in cell diffusion and membrane flickering; however, RBC diffusion can practically be neglected in the current simulations in comparison to RBC transport induced by the flow due to flow rates being sufficiently large, resulting in a high Péclet number. Consequently, even the trajectories with a non-integer lane-swapping frequency are expected to be deterministic for the fixed simulation conditions. In experiments, there also exist a number of uncertainties (e.g., cell properties, flow control, device fabrication limits) which may affect the trajectory of a cell in the device. The inherent variability in RBC properties can perhaps be least controlled. However, the example of experimental results for section 11 of the thick device in Fig. 2(b), which consists of 69 well-aligned RBC trajectories (see Supplementary Figure S4), shows a consistent pattern of traversing 4 or 5 obstacles before performing a lane-swap. This indicates that the trajectories are not random, as otherwise a much broader variety of lane-swapping patterns would be observed, and that the geometry of section 11 and the corresponding flow are not very sensitive to ‘moderate’ variations in RBC properties. This complex yet deterministic nature of trajectories can be explained by an interplay between RBC dynamics (e.g., flow-rotation, deformation) and local flow mainly determined by the periodic geometry of the DLD device. Thus, a mismatch between RBC motion and periodic geometry may result in several different cell states (e.g., position, orientation) after a lane-swap, which will in turn decide the number of pillars traversed before the next swap and indeed the lane-swapping pattern thereafter.

**Figure 3:**
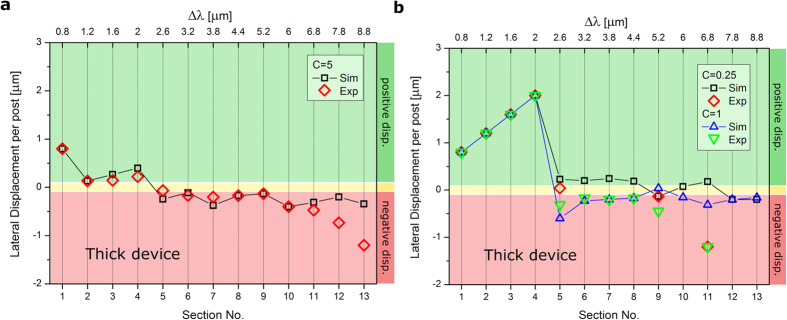
The average lateral displacement per post encounter *l* of RBCs in each individual section of the thick device at various viscosity contrasts *C* = *η*_*i*_/*η*_*o*_. (**a**) Data for the thick device at *C* = 5. Experimental values agree well with the simulated values and the transition to zig-zag modes occurs between sections 1 and 2. (**b**) Data for the thick device at *C* = 1 and *C* = 0.25. Experimental and simulation trajectories at both viscosity contrasts undergo a transition from displacement to zig-zag modes between sections 4 and 5, which is later than found at a physiological value of *C* = 5.

By plotting the change in *l*_*i*_ as RBCs travel through the entire device, we are able to see the transitions between different sorting modes. The average lateral displacement *l*_*i*_ per post encounter for each individual section of the thick device is shown in Fig. 3(a) and the number of RBC trajectories used to calculate each *l* value can be found in the Supplementary Tables S2–S6. These data allow us to draw several important conclusions. Firstly, we find very good agreement between simulated and experimental trajectories for a viscosity contrast of *C* = 5. The only real deviations occur in the final few sections of the device, where the experimental trajectories are observed to undergo a more negative lateral displacement due to disturbances of the flow field when in the vicinity of the outlet; an effect we see consistently throughout the course of this work. The close agreement suggests that the simulation techniques adequately capture the hydrodynamic effects and RBC deformation and dynamics. Secondly, the transition from displacement to zig-zag mode occurs at the beginning of the device, between sections 1 and 2. After this transition the RBC transit modes become gradually negative as the lateral row shift Δ*λ* increases with section number. The increase in Δ*λ* promotes positive displacement due to geometry, however there is also a higher likelihood of lane-swapping events due to the increase of critical radius. These zig-zag modes with negative lateral displacement per post are not very far from the neutral mode, however they present a key manifestation of the transit behavior of deformable particles unavailable to hard spheres, and are due to the dynamic properties of RBCs with the responsible mechanisms postulated later in the discussion section. Finally, we note that the additive effect of some sections may cancel each other out, making their serial use in one device inefficient for separation.

### DLD transit modes in the thin device

**Figure 4:**
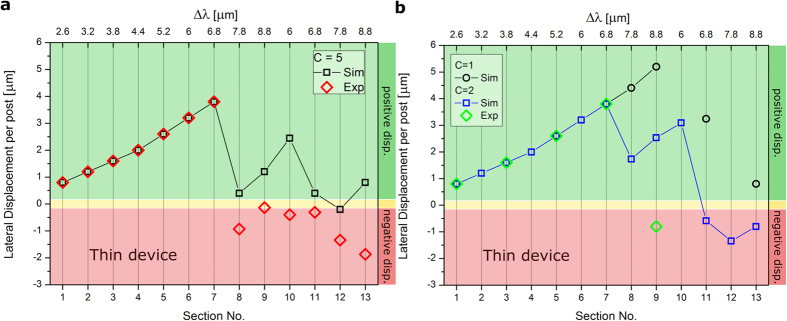
The average lateral displacement per post encounter *l* of RBCs in each individual section of thin device at various viscosity contrasts *C* = *η*_*i*_/*η*_*o*_. (**a**) Data for the thin device at physiological viscosity contrast *C* = 5. Transition to zig-zag modes is the same in experiments and simulations, occurring between sections 7 and 8. However, evolution of zig-zag modes with section number shows discrepancies due to inherent variability in RBC size. (**b**) Data for the thin device at viscosity contrasts *C* = 1 and *C* = 2. Transition to zig-zag modes at contrast *C* = 2 occurs between sections 7 and 8 for simulations and experiments, whereas the transition at *C* = 1 occurs in section 10 for the simulations. Again, variability between experimental and simulation results can be attributed to the inherent variability in RBC diameter.

The thin device inhibits RBC orientation in flow, forcing the cell to align with the device plane. The highly constrained environment limits the range of dynamics available to the RBCs and therefore, the results should be closer to those predicted for hard spheres. Figure 4(a) shows the average lateral displacement of a RBC per post encounter for each section of the thin device. For a viscosity contrast *C* = 5 in sections 1–7 an excellent agreement between the experimental and simulated values is observed, showing strong positive lateral displacement. Only in section 6 does lane-swapping begin to occur. However, these events are infrequent and the video frame is too narrow to capture two successive lane-swapping events for the same trajectory, making it difficult to obtain a period for a zig-zag mode. Similarly, it is difficult to run simulations long enough to accurately capture several lane-swapping events in section 6. Ultimately, we see a strong positive displacement in sections 1–7 of the thin device at *C* = 5. In the following sections, lane-swapping events occur frequently and RBCs travel in well-established zig-zag modes in the simulated and experimental trajectories. This transition from displacement dominated trajectories to lane-swapping dominated trajectories in section 8 agrees relatively well with the empirically predicted critical radius of *R_c_* *=* 3.02 μm for hard spheres^8^, considering that the maximum RBC radius is about 4.00 μm and it will often be less than this due to deformation in the flow. It is also important to notice the discrepancy between experimental and simulated trajectories after the transition to zig-zag modes in Fig. 4(a), which will be discussed below.

A sharp transition from pure positive displacement modes to zig-zag modes occurs in section 8 of the device for *C* = 5 (Fig. 4(a)). It is in this section and subsequent ones that we begin to see less agreement between simulations and experiments. Specifically, simulated trajectories undergo less negative lateral displacement due to a smaller frequency of lane-swapping events. Furthermore, we see increasingly positive zig-zag modes in sections 8–10 in simulations, which is due to the increasing value of Δ*λ* in subsequent sections causing an increased positive lateral displacement but a relatively low increase in the frequency of lane-swapping events. Larger deviations between experimental and simulation results are not unexpected when the confinement of the RBC is increased in a device, because the forced orientation of the RBC in the thin device amplifies the effects of size differences between simulated and sample RBCs. The diameter of real RBCs varies in the range between 6 μm and 9 μm, which is approximately a 10–15% deviation from the simulation value of 8 μm. The possible differences in size are on the same order of magnitude as the change in critical radius for successive sections of the thin device.

**Figure 5:**
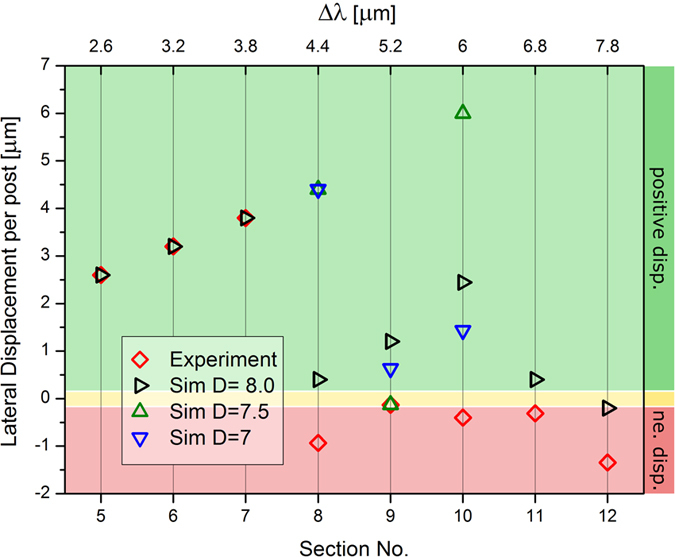
Sensitivity of the thin device to RBC size. The average lateral displacement per post encounter *l* for individual sections of the thin device. Several RBC diameters were considered to confirm that the later sections of the thin device are quite sensitive to moderate changes in the RBC size. Several simulations for the RBCs with diameters 7, 7.5 and 8 μm have been performed.

To investigate the effect of RBC size in more detail, we performed several simulations for RBCs shown in Fig. 5 with different diameters at *C* = 5. The simulation results for different RBC sizes indicate that the discrepancies between experimental and simulated trajectories are likely to come from the variations in size of real RBCs. These results also confirm that the later sections of the thin device are very sensitive to moderate changes in the RBC size. Furthermore, a close quantitative agreement between experiments and simulations in Fig. 5 is difficult to obtain, because the experimental data points correspond to the averages of several trajectories of RBCs with potentially different sizes. In fact, a number of experimental trajectories were not taken into account, because they could not be aligned following our alignment criteria. The number of disregarded trajectories for the thin device was considerably larger than that for the thick device, as can be seen in Supplementary Tables S3 and S5, showing a decrease in the fraction of accepted trajectories from the videos in these sections. The thick device appears less sensitive to RBC size differences, since the effective RBC size corresponds to the cell thickness rather than to the RBC diameter due to its orientation in flow. The simulation results also illustrate that we are able to confidently check device sensitivity to values which are hard to measure and consistently manipulate in a lab setting, such as a variable cell size.

### Viscosity-contrast-based sorting

The viscosity contrast between the intra- and extra-cellular fluids in simulation and experiment discussed so far is close to the value of *C* = *η*_*i*_/*η*_*O*_ = 5 for physiological conditions in real blood. However, it is well established that the viscosity contrast is an important factor in RBC dynamics in shear flow, as it leads to a transition from tank-treading to tumbling with increasing viscosity^20^,^21^. By altering the viscosity contrast, we demonstrate the importance of RBC dynamics within the device when attempting to use DLD as a technique for RBC separation. In addition to viscosity contrast *C* = 5 between the intra- and extra-cellular fluids, simulations and experiments were carried out at *C* = 1 and *C* = 0.25 for the thick device, and *C* = 2 for the thin device. In the experiments, the viscosity of the outer fluid is increased by adding dextran at various concentrations. Note that the different choice of *C* = 1 and *C* = 2 for the thick and thin devices, respectively, is due to pronounced adsorption (or sticking) of RBCs to the upper and lower walls, which occurs in the confined environment of the thin device for the higher dextran concentrations required to reach a viscosity contrast less than *C* = 2.

The lateral displacement per post *l* of the trajectories under these additional viscosity contrast conditions are shown in Figs 3(b) and 4(b). Simulations were first used to predict which sections display interesting behavior, and these sections were then investigated experimentally in order to validate results. For both *C* = 1 and *C* = 0.25 in sections 1–4 of the thick device, the change in viscosity contrast completely inhibits lane-swapping events. This substantial shift away from behavior in the physiological case of *C* = 5 may be attributed to a change in RBC dynamics which will be discussed later. In subsequent sections, there is a transition to almost neutral zig-zag modes, with a gradually increasing tendency to adopt an average negative lateral displacement towards the later sections of the device. In the sections following the transition, the zig-zag modes at *C* = 0.25 are slightly more positive than those for the *C* = 1, but the *l* values for both cases converge in the last two sections of the device. This suggests a small difference in dynamic behavior which is only relevant in deciding the lane swapping frequency in the middle sections of the device. Generally, we see good agreement between experimental and simulated results, the main differences occur in the later sections of the device which we attribute to a distortion of the flow field when in close proximity to the device outlet. The difference between the trajectories in the early sections at viscosity contrast *C* = 5 compared with those at *C* = 1 and *C* = 0.25 demonstrates the importance of viscosity contrast for RBC sorting since it may dramatically alter the transit modes.

Figure 4(b) shows that the viscosity contrast also plays an important role in the transit of RBCs through the thin device. For a viscosity contrast of *C* = 1, the simulated RBC trajectories undergo a transition from the displacement mode to zig-zag mode only at section 10, while for *C* = 5 the transition occurs earlier, at section 8. Additionally, for the intermediate viscosity contrast of *C* = 2, the transition to zig-zag modes occurs in the same section as for *C* = 5. Well-defined zig-zag modes follow the transition, with a region of positive zig-zag modes in sections 8–10. The *l* values in sections 8–10 are more positive than for the physiological contrast *C* = 5, and represent a behavior intermediate between the *C* = 5 and simulated *C* = 1 values. Generally, we see good agreement between simulated and experimental results, as again we have to consider the potential effects of RBC-size variations which we already presented in Fig. 5.

There are also differences in the average lateral displacement per post encounter for the viscosity contrasts *C* = 1 and *C* = 2–5 in later sections of the device, which are especially pronounced in section 11. These results suggest that changes in RBC dynamics and deformation are still relevant in the thin device and that their effects are most pronounced on well-established zig-zag modes. As a conclusion, our results suggest that the viscosity contrast could be used as a targeted separation parameter by itself without other changes in RBC properties.

### RBC dynamics in DLDs

The dependence of the transit modes of RBCs traveling through DLD obstacle arrays on viscosity contrast has revealed the importance of RBC dynamics. Single RBCs in shear flow have been shown experimentally to tumble at low shear rates and tank-tread at high shear rates^18^,^19^,^22^,^23^. Note that all these experiments have been performed under the conditions where the viscosity of suspending media was larger than that of the RBC cytosol. However, recent experiments^20^ and simulations^21^ indicate that the physiological viscosity contrast of 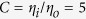 suppresses the tank-treading motion of RBCs, leading to the preference for RBC tumbling. In case of 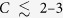, RBC membrane tank-treading is possible and the transition between tumbling and tank-treading for an increasing shear rate is attributed to the existence of a RBC minimum energy state, related to the weakly anisotropic shape of the spectrin network, such that the RBC has to exceed a certain energy barrier in order to transit to the tank-treading motion^23^. Moreover, recent shear-flow experiments^19^ have identified another dynamic state, RBC rolling, which occurs within the range of shear rates between RBC tumbling and tank-treading states.

**Figure 6:**
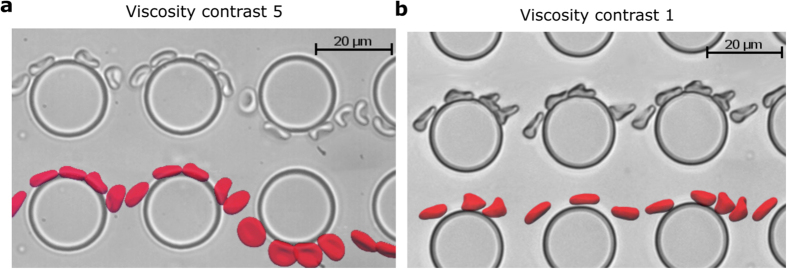
Stroboscopic images of RBCs in section 2, taken from simulations and experiments. **(a)** RBC lane swapping is promoted by tumbling when 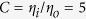. **(b)** Tank-treading type dynamics occurs at *C* = 1 and the RBC favors the displacement mode.

The main difference in RBC dynamics for the cases of *C* = 0.25–1 and *C* = 5 is the preference of tank-treading motion and tumbling dynamics, respectively. Figure 6 shows snapshots of simulated and experimentally observed RBCs in section 2 of the thick device at viscosity contracts *C* = 1 and *C* = 5. In addition to demonstrating the accuracy of the simulated RBC dynamics in comparison to the corresponding experimental data, Fig. 6 demonstrates the preferred transit modes of RBCs exhibiting different dynamic behavior. The tumbling and rolling motion, which occurs at the physiological viscosity contrast, favor a zig-zag transit mode. Conversely, the tank-treading dynamics at viscosity contrast *C* = 1 noticeably inhibits lane swapping and the membrane deforms considerably (see trilobe RBC shapes in Fig. 6(b)) due to the shear forces experienced in the local vicinity of obstacles. Also, we observe in Fig. 6(a) that RBC tumbling motion directly precedes and follows lane-swapping events, indicating that tumbling dynamics plays an important role in determining the lane-swapping frequency of zig-zag modes. In comparison to tumbling motion, the tank-treading RBC seen in Fig. 6(b) is subject to local shear-flow alignment, which suppresses cell tumbling and therefore, swapping of lanes. This fact is consistent with the simulation data in Fig. 3, where the transition to zig-zag modes occurs at a later section for *C* = 1 and *C* = 0.25 in comparison with *C* = 5. The results for *C* = 0.25 and *C* = 1 are very similar, and this can be attributed to the fact that the only difference in RBC dynamics will be an increase in the frequency of tank-treading at *C* = 0.25 due to the reduced internal viscosity. This minor change in dynamics is reflected in the small changes in *l* values between the two cases.

Another important aspect which influences the transit of RBCs through a DLD is related to hydrodynamic interactions of deformable particles with walls and obstacles. It is well known that deformable particles (including RBCs) in flow near a wall are subject to a lift force driving them away from the wall^24^,^25^,^26^. Even though the lift force would depend on particle properties (e.g., rigidity, viscosity contrast) and its dynamics (e.g., tank-treading or tumbling, inclination angle), it is well established that the lift force is stronger on a tank-treading RBC in comparison to a tumbling cell. Thus, the lift force on a RBC from the pillars in the thick device may inhibit the transition to zig-zag modes in early sections for *C* = 1 and *C* = 0.25 in comparison with *C* = 5.

## Discussion and Conclusions

We have presented a detailed comparison of experimental and simulated results for RBC transit through chirped DLD devices under various conditions. The behavior of RBCs in DLD sorting devices is different from that of rigid spheres in two key ways. The first distinction is that a RBC has many more transit modes available than a rigid particle due to the complex interplay between hydrodynamic interactions with obstacles (i.e., lift force), and RBC orientation and deformation. The second distinction follows directly from the first: given that a RBC’s varying orientation and deformation depends on features such as viscosity contrast and deformability, changing these parameters will result in transitions to different transit modes. For instance, the preference for RBC tumbling at *C* = 5 enables the transition from pure displacement to zig-zag mode in section 2 of the thick device, while the tank-treading motion of a RBC membrane at *C* = 1 and *C* = 0.25 delays this transition up to section 5. The main difference between these two cases arises from a well-documented hydrodynamic interaction of cells with a surface which is called the lift force and experienced by deformable particles in flow next to walls^24^,^25^,^26^. The lift force is stronger on a tank-treading cell in comparison to that performing tumbling dynamics, and pushes a RBC away from a pillar resulting in a delayed transition from the displacement to zigzag mode. Note that these hydrodynamic effects do not exist for rigid spheres when inertial effects can be neglected (i.e., nearly zero Reynolds number), which is the case for our experimental and simulation conditions.

Another effect, which can be important for deciding particle trajectory in DLDs, is related to direct particle-post collisions. Bowman *et al*.^27^ presented a model based on the irreversibility of particle trajectory due to its collision with a post, which has rationalized well the traversal of drops in a gravity-driven DLD. In that study, the drops did not experience significant deformation since very low capillary numbers were employed, and therefore, the deformability effect can be excluded. However, it remains unclear whether the particle-post volume-exclusion effect is relevant for our DLD geometry and whether its contribution would be strong enough in comparison with the dynamics and deformability effects for RBCs described above.

For a thick device, which allows complete freedom in RBC orientation, an excellent quantitative agreement between experiments and simulations with a viscosity contrast *C* = 5 between the intra- and extra-cellular fluids has been observed. As already mentioned for viscosity contrasts *C* = 0.25–1, a change in RBC dynamics resulted in the inhibition of lane-swapping events in early sections of the device. Simulation results for the thin device at viscosity contrasts *C* = 2–5 show a good quantitative agreement for the displacement modes located in the first 7 sections of the device. Discrepancies between simulation and experiment following the transition to zig-zag modes in sections 8 and onwards are explained by the inherent experimental variability in RBC size, a characteristic which the thin device is especially sensitive to. Indeed, changing simulated RBC size within the range of real RBC sizes or varying the viscosity contrast dramatically alters the average lateral displacement per post of RBCs in sections 8–10, which immediately follow the transition from displacement to zig-zag modes. Furthermore, simulations with a viscosity contrast *C* = 1 in the thin device display a similar inhibition of lane swapping seen in the thick device in comparison with the case of *C* = 5 or *C* = 2, as the transition to zig-zag modes shifts from section 8 to section 10.

Based on our observations we can make several suggestions for device design and optimization, and possible future sorting schemes. Foremost, we see that neutral zig-zag modes and perfect displacement modes make only a small fraction of the possible cell transit modes. In order to design an optimal chirped device for sorting deformable particles, it will be necessary to consider the net displacement resulting from a range of negative, neutral and positive transit modes. Our results for different viscosity contrasts suggest that carefully selecting the viscosity of the suspending medium may allow sorting based on the viscosity of particles’ intra-cellular fluid. A sorting scheme of this kind would be performed best in a *thick* device which allows exploration of the full range of the particle dynamics induced by the viscosity contrast. For instance, two types of particles which differ only in their internal viscosity may be separated by tuning the viscosity of the suspending medium such that each particle’s dynamics favors a different transit mode.

The other potential for such devices is elasticity-based sorting, which has been partially explored in recent experiments^13^. Based on the knowledge about RBC dynamics in shear flow^18^,^19^,^20^,^23^, it is plausible to expect DLD devices to be well suited for elasticity-based sorting of RBCs, which might be relevant in several blood diseases. Here, again the transitions between tumbling, rolling, and tank-treading motions^19^ might be exploited; however, they appear to be suppressed by a high enough viscosity contrast^20^,^21^ favoring tumbling motion. Therefore, we expect that elasticity-based sorting of cells in the thick device would be achieved best at a viscosity contrast smaller than about 2–3.

Elasticity-based sorting of RBCs should theoretically be also possible in the thin device. The simulation results in Fig. 4 for the thin device indicate that in the later sections there exist differences between the simulation results for viscosity contrasts *C* = 2–5 and *C* = 1. These differences must come from RBC deformability in flow (here due to different fluid viscosities) and the later sections of the thin device are very sensitive to slight changes in effective RBC size which may occur due to cell deformation in flow. In practice however, as we have seen in Fig. 5, the trajectories in sections immediately following the zig-zag mode transition are somewhat unpredictable due to inherent variability in RBC sizes, so it is unlikely that the device would be able to distinguish between small changes in size and small changes in deformability. The deformation effect has been nicely illustrated in the recent simulation study^16^, where the effect of flow rate on the transit mode of a RBC in a similar thin device has been explored. An increase of flow rate in the device leads to stronger RBC deformations and change of the transition between the displacement and zig-zag modes. Thus, a proper tuning of the flow rate would be necessary for elasticity-based sorting of RBCs in the thin DLD. Returning to size-based sorting, we see that a thin device suppresses the variability in RBC dynamics and is consequently more sensitive to variations in cell size. As such, thin chirped devices look best suited for sorting RBCs based on their size.

For rigid spheres, there exist empirical relations between device geometry and the critical particle size where trajectories switch from the displacement to zig-zag mode^28^,^29^. This relation is possible due to the fact that there are only two available transit modes and a particle’s preference for each depends solely on its size relative to the characteristic length scale of the device geometry. However, as demonstrated here for deformable particles, the transition between displacement and zig-zag modes and the presence of additional modes are much harder to predict, because the behavior depends on a complex combination of several additional variables such as particle elasticity, viscosity contrast, and flow speed. Consequently, a simple empirical relation for all these variables seems unlikely to be found. Also, any modeling simplification (e.g., omission of some cell properties) would not lead to predictive results for the transit in DLDs, and therefore it is necessary to use quantitatively reliable fluid simulation techniques which properly account for hydrodynamic effects, and particle models which can accurately capture the mechanical properties of deformable particles such as elasticity, the viscosity ratio between extracellular and intracellular fluids, and cell morphology.

In conclusion, we have demonstrated that the complex interplay between the dynamic behavior of deformable particles and the hydrodynamic interactions they experience near obstacles give rise to a much richer behavior in DLD devices than that found for rigid spheres. Still, the transit modes are not random but deterministic, and are determined by fluid flow and the mechanical properties of deformable particles. Thus, the combination of predictive simulations and experiments becomes a powerful tool for the design of novel sorting schemes which employ dynamic behavior and intrinsic mechanical characteristics of bioparticles as a quantitative separation parameter or biomarker. Such sorting schemes are not restricted only to blood related diseases and disorders (e.g., malaria, sickle-cell anemia), but can also be employed in many other areas including bacteriology, parasitology, and oncology. In addition, label-free microfluidic sorting of bioparticles by their dynamical properties offers significant improvements over conventional techniques, such as fluorescence- and magnetic-activated cell sorting^1^,^2^, by making sample handling easier and reducing costs. Finally, our work demonstrates the importance of reliable predictive simulation approaches for the development of purpose-specific microdevices, since simulations can provide a better understanding of dynamic behavior of bioparticles in microfluidics and lead to significant design optimizations.

## Methods

### Simulation techniques

To represent the suspending fluid, we employ a mesoscale hydrodynamic simulation approach which is a variation of the smoothed dissipative particle dynamics (SDPD) method^30^, adapted to conserve angular momentum^31^. The RBC membrane is modeled as a triangulated network of springs^32^,^33^,^34^, whose vertices are coupled to the fluid via frictional forces.

The obstacle array is simulated using a 3D domain enclosing a single-column obstacle with its axis in the z direction, perpendicular to the roof and floor of the domain box. Efficient representation of an infinite bumper array environment is achieved with periodic boundary conditions in the *x* and *y* directions (along and perpendicular to the flow direction) and a shift in the *y* direction for each boundary-crossing event in the *x* direction, as depicted in Supplementary Figure S6. The flow was driven by a body force applied to each solvent particle in the x direction, which mimics the pressure drop used in experiments. No cross flow in y direction in this periodic configuration is enforced by applying an adaptive force in y direction to mimic side walls of a real DLD device. The floor, ceiling, and pillar walls of the DLD device are modeled by a layer of frozen particles which share the same equilibrium structure as the suspending fluid. In order to prevent particles from penetrating the walls and mixing of the intra-cellular fluid and suspending medium, RBC vertices and fluid particles are subject to bounce-back reflections at walls and RBC membrane. Finally, in order to ensure no-slip boundary conditions at solid walls, an adaptive tangential force is applied to fluid particles next to impenetrable surfaces. For more simulation details see Supplementary Information.

### Experimental methods

Devices were fabricated by replica molding, using the same method and equipment as in previous work by Beech *et al*.^13^, and the post-manufacture details are outlined in Supplementary Information. The experimental devices consist of 13 consecutive sections of obstacle arrays, which are differentiated by different lateral shifts Δ*λ*. The geometry of a DLD device^5^,^13^ is defined by the post diameter *D* = 20 μm, lateral center-to-center spacing between posts *λ* = 32 μm, lateral shift in successive pillar rows Δ*λ*, and the height between the two enclosing plates *H*, as shown in Supplementary Figure S1. Two devices with this geometry are used and can be distinguished by the distance between the top and bottom plates covering the obstacle arrays. One device has a height of *H* = 11 μm, which is larger than a RBC diameter of about 8 μm, while the other device had *H* = 4 μm, which is smaller than the RBC diameter but larger than the RBC thickness of about 2–3 μm.

The study was approved by the Review Board of Lund University and performed in accordance with the applicable guidelines and regulations. Blood was taken from healthy volunteers (informed consent was obtained from all subjects) via finger pricking and diluted in an autoMACS™ buffer to achieve a viscosity contrast *C* = 5 environment. A Dextran-500 (#700013-096, VWR International LLC, PA, USA) solute was added at concentrations of 11%, 4.5%, and 1% in order to perform experiments at viscosity contrasts of *C* = 0.25, 1, and 2, respectively. The solutions were driven through the devices at a pressure drop of 22 mbar. Running the simulations at a comparable pressure drop, we calculate the average fluid velocity to be about 0.5 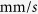 for the thick device and 0.12 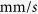 in the thin device. These values correspond to the physiological values for blood flow in capillaries.

## Additional Information

**How to cite this article**: Henry, E. *et al*. Sorting cells by their dynamical properties. *Sci. Rep.* **6**, 34375; doi: 10.1038/srep34375 (2016).

## Supplementary Material

Supplementary Information

Supplementary Video

Supplementary Video

Supplementary Video

Supplementary Video

Supplementary Video
