# Supplementary material for: Sorting cells by their dynamical properties
Source: Sci Rep. 2016 Oct 6;6:34375. doi: 10.1038/srep34375 (PMC5052630; doi:10.1038/srep34375)
Supplement: Supplementary Information [file srep34375-s1.pdf]

SUPPLEMENTARY INFORMATION

Sorting cells by their dynamical properties

Ewan Henry[1], Stefan H. Holm[2], Zunmin Zhang[1], Jason P. Beech[2], Jonas O. Tegenfeldt[2], Dmitry A. Fedosov[1], and Gerhard Gompper[1]

[1] *Theoretical Soft Matter and Biophysics, Institute of Complex Systems and Institute for Advanced Simulation, Forschungszentrum Jülich, 52425 Jülich, Germany; Email: d.fedosov@fz-juelich.de, g.gompper@fz-juelich.de*

[2] *Division of Solid State Physics, NanoLund, Lund University, PO Box 118, S-221 00 Lund, Sweden; Email: jonas.tegenfeldt@ftf.lth.se*

Contents

1 Experimental materials and methods **1**
1.1 Device fabrication and design . . . 1
1.2 Sample preparation . . . 2
1.3 Data acquisition and analysis . . . 3

2 Numerical methods **5**
2.1 Fluid simulation - mesoscale hydrodynamics . . . 5
2.2 Red blood cell model - triangulated surfaces . . . 8
2.3 Simulation set-up and boundary conditions . . . 9

3 Supplementary videos **11**
3.1 Viscosity contrast $C = 5$ . . . 11
3.2 Viscosity contrast $C = 1$ . . . 11

1 Experimental materials and methods

1.1 Device fabrication and design

Devices were fabricated by replica molding, using the same method and equipment used in previous work by Beech *et al.* [1]. Adhesion of blood cells to the inner surface of our devices was reduced by formation of a polymer brush on the polydimethylsiloxane (PDMS). Immediately after O_2 plasma treatment and bonding, devices were filled with 0.2% PLL(20)-g[3.5]-PEG(2) (SuSoS AG, Dübendorf, Switzerland) and rinsed after 20 minutes with autoMACS™.

The experimental device has been designed to sort particles of different sizes, with successive sections within the device corresponding to different critical radii [2]. The device geometry [1,2] is defined by the post diameter $D = 20\,\mu m$, lateral center-to-center spacing between posts $\lambda = 32\,\mu m$, lateral shift in successive pillar rows $\Delta\lambda$, and the height between the two enclosing plates H, as shown in Supplementary Figure S1. The lateral shift can conveniently be defined as a fraction ϵ of the post spacing $\Delta\lambda = \epsilon\lambda$. The experimental device consists of 13 consecutive sections of obstacle arrays, which are differentiated by the different lateral shifts $\Delta\lambda$ between successive rows as summarized in Supplementary Table S1. We note that some of the sections have row-shift fractions ϵ that are equivalent to M/N, where M and N are integers and $M \neq 1$, i.e. we have a "non-integer" row-shift fraction. This is due to limitations in the photomask fabricating process (a manufacturing grid of 200nm), which means that in many cases the desired integer row-shift fractions are not possible, in which case the nearest non-integer row-shift fraction is used. While non-integer row-shift fractions have been shown to give rise to two additional zig-zag modes with accompanying critical sizes [3,4], in the present study these modes are not observed.

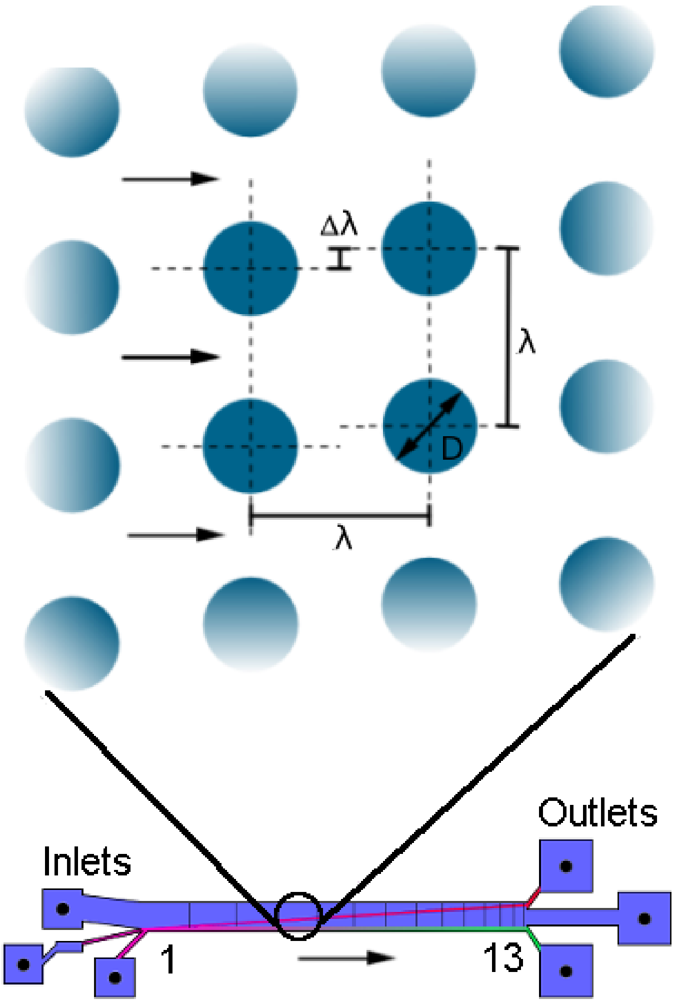

Supplementary Figure S1: (top) A close-up bird's eye view of the obstacle array. **(bottom)** Schematic of the sequential device with 13 sections, each with different $\Delta\lambda$. The flow direction is indicated by the arrows. Solution from the inlets on the left is carried by the flow within DLD to the right, where the particles of different sizes are collected at various outlets.

Two sequential devices have been used, distinguished by the distance between the top and bottom plates covering the obstacle arrays. One device had a height of $H = 11\,\mu\text{m}$, which is larger than a RBC diameter of about $8\,\mu\text{m}$, while the other device had $H = 4\,\mu\text{m}$, which is smaller than the RBC diameter but larger than the RBC thickness of about $2-3\,\mu\text{m}$. We will refer to these two devices as 'thick' and 'thin' DLDs, respectively.

Several inlets and outlets were employed to control flow and sample input/output, see Supplementary Figure S1. Both devices had one large fluid buffer inlet and one small buffer inlet to minimize the effects of walls and to create a well defined starting position to which total displacement can be compared. The sample inlet channel has been placed between the two buffer inlets and included a filter to remove any large particles which could cause clogging. A pressure gradient was used to drive the flow. Outlets were kept at atmospheric pressure and the overpressure at the inlets was maintained using a MFCS-4C pressure controller (Fluigent, Paris, France). Flow in the devices was driven by a pressure drop of 22 mbar between the buffer inlets and outlets.

1.2 Sample preparation

Blood was extracted from healthy volunteers via finger pricking. As mentioned in the paper, measurements were conducted both at a viscosity contrast of 5 and at a viscosity contrast of 1 between the cytosol and the surrounding

Section	$\Delta\lambda$/µm	ϵ	$\frac{1}{\epsilon}$	Rows	R_c/µm
1	0.8	0.025	40	200	1.43
2	1.2	0.0375	80/3	130	1.74
3	1.6	0.05	20	100	2.0
4	2.0	0.0625	16	80	2.22
5	2.6	0.0813	160/13	60	2.52
6	3.2	0.1	10	50	2.78
7	3.8	0.1188	160/19	40	3.02
8	4.4	0.1375	80/11	35	3.24
9	5.2	0.1625	80/13	30	3.51
10	6.0	0.1875	16/3	25	3.76
11	6.8	0.2125	80/17	20	4.0
12	7.8	0.2438	160/39	20	4.27
13	8.8	0.275	40/11	15	4.52

Supplementary Table S1: Parameters defining the obstacle array geometry for each section of the DLD device. $\Delta\lambda$ is the lateral shift between successive rows for each section, $\epsilon = \Delta\lambda/\lambda$ is the section's shift fraction, and R_c is the critical particle size [5, 6]. The fourth column in the table specifies the number of rows in each section.

buffer. For high viscosity contrast measurements, the cells were suspended in autoMACS™ running buffer (Miltenyi Biotech, Auburn, CA). This solution (pH 7.2) contains phosphate buffered saline (PBS) supplemented with 0.5% bovine serum albumin (BSA), 2 mM EDTA, and 0.09% azide. It acts to suppress blood clotting and is isotonic so it does not affect the cell shape due to a changed osmotic pressure. For low viscosity contrast measurements the autoMACS™ running buffer was supplemented with Dextran-500 (# 700013-096, VWR International LLC, PA, USA). To measure the viscosity (see Supplementary Figure S2), Ubbelohde viscometers were used (UBBEL Visco,Paragon Scientific Ltd, UK.). The temperature ranged between 21.8-22.°C, which is similar to the measurements conducted with cells in the DLD device.

Dextran is widely used to change the viscosity of various samples. It is a neutral polysaccharide, i.e. the pH or salt concentration of the solution does not affect the resulting viscosity. In order to minimize the change to the osmotic pressure across the cell membrane, this larger Dextran molecule with an average molecular weight of 500 kDa was chosen. At a concentration of 11% the change in osmotic pressure is 0.22mM which is negligible compared to isotonic blood osmolarity of 300mM. Furthermore, microscopic examinations also confirmed that the low viscosity contrast samples did not visibly differ compared to the high viscosity contrast samples.

1.3 Data acquisition and analysis

All images were taken through an inverted Nikon Eclipse TE2000-U microscope (Nikon Corporation, Tokyo, Japan). High-speed images were taken using an EoSens mini MC-1370 camera (Mikrotron GmbH, Unterschleissheim, Germany). In all other cases an Andor iXon EMCCD camera (Andor Technology, Belfast, Northern Ireland) or Hamamatsu Orca Flash 4.0 (Hamamatsu, Shizuoka Pref., Japan) were used. Throughout the course of experiments, the dynamics and displacement of single RBCs in different device sections have been monitored.

The simultaneous transit of many RBCs through each section of the DLD device has been recorded to video using the microscope and camera set-ups previously described. Trajectories of individual RBCs were extracted from these recordings using the particle-tracking application MOSAIC in the image processing suite ImageJ [7]. Particle detection has been improved by subtracting a median average of all frames from the entire video and removing the background.

After extracting the sets of trajectories for different device sections from the video recordings, a pre-screening procedure has been performed. In the pre-screening process, trajectories which are too short or where direct interactions between RBCs have been detected, were discarded. As a rule of thumb, trajectories were considered to be too short if they were much shorter than the video domain, corresponding to RBC traversal over only a few pillars in the device. The short trajectories might be present at the beginning or the end of videos and also due to a detection failure if at some point video contrast becomes insufficient for tracking. The trajectories with direct interactions between RBCs

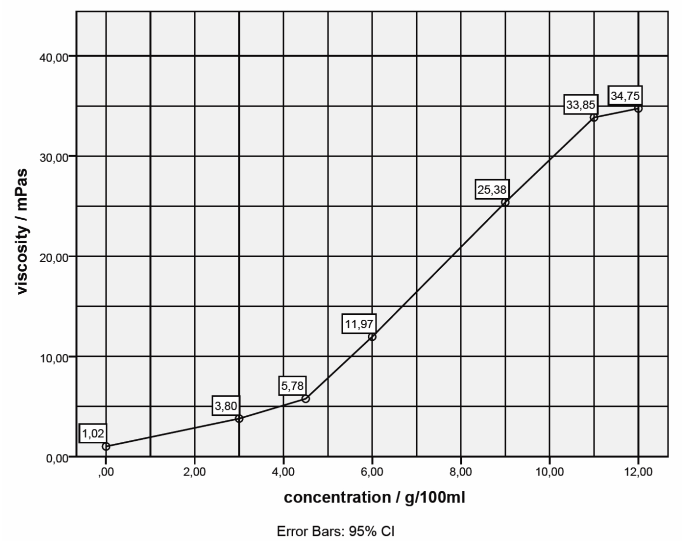

Supplementary Figure S2: Viscosity measurement of different dextran-500 concentrations in autoMACS. In order to achieve a viscosity contrasts of $C = 2, 1$ and 0.25 between the medium and the cytosol, the suspending medium viscosity needed to be increased to two, five and twenty five times that of normal autoMACS buffer viscosity.

correspond to those where collisions between RBCs were detected. These trajectories were also excluded from further analysis, since such situation has not been considered in simulations.

In order to establish a common pattern followed by the RBCs in a given section of the device, all pre-screened trajectories were superimposed on top of one another and averaged into a single trajectory. Optimal positioning of trajectories for superposition has been found by the minimization of the Hausdorff distance between each member in the set of trajectories. The Hausdorff distance $d_H(X, Y)$ between two sets (X, Y) of points in 2D Cartesian coordinates is defined as

$$d_H(X, Y) = \max\{ \sup_{x \in X} \inf_{y \in Y} d(x, y), \sup_{y \in Y} \inf_{x \in X} d(x, y) \}, \quad (1)$$

where *sup* denotes the supremum and inf the infimum. $d_H(X, Y)$ can be thought of conceptually as the largest of all distances from a point in one set to the closest point in a second set. This method for the alignment of trajectories is very similar to techniques used in image-recognition algorithms [8]. In our algorithm, we have fixed a base trajectory in place, while a second trajectory traverses an overlaid grid to find the position with a minimum Hausdorff distance to the former trajectory. Additional refinement of the RBC trajectories has been done during this alignment stage, where some outliers were removed if computed d_H of a trajectory was larger than one standard deviation from the mean d_H value of all trajectories in the given section. These outliers can arise for multiple reasons, such as the presence of slightly different transit modes within one section (see Supplementary Figure S4), possible blockage between two posts (see Supplementary Figure S5), or even due to missing posts which might be due to some problems in device fabrication.

A summary of the RBC trajectory refinement for each device is shown in Supplementary Tables S2, S3, S4, and S5, where the numbers of total, accepted, and rejected trajectories are given for all experiments. Supplementary Figure S3 presents visually the fraction of accepted trajectories for the thick and thin devices at $C = 5$. Notice that the sections with lowest trajectory acceptance are those where the transition between displacement and zigzag modes is occurring, or where extremely negative zigzag modes with slight variations in row-swapping frequency result in large differences in lateral displacement per pillar. The possibility of different transit modes within a single section is illustrated in Supplementary Figure S4 for the section 11 of the thick device. 69 aligned trajectories yield a main average trajectory in this section with a displacement-zigzag pattern {5,4,5,4,4,5,4}, while 17 rejected trajectories seem to correspond to two other displacement-zigzag patterns {5,4,4,4,5} or {5,4,5,5,4}. The differences can arise from natural variations in RBC properties indicating that this section might be sensitive to them. However, it is also possible that the recorded trajectories are too short to properly capture the period of transit mode in section 11 and the displacement-zigzag

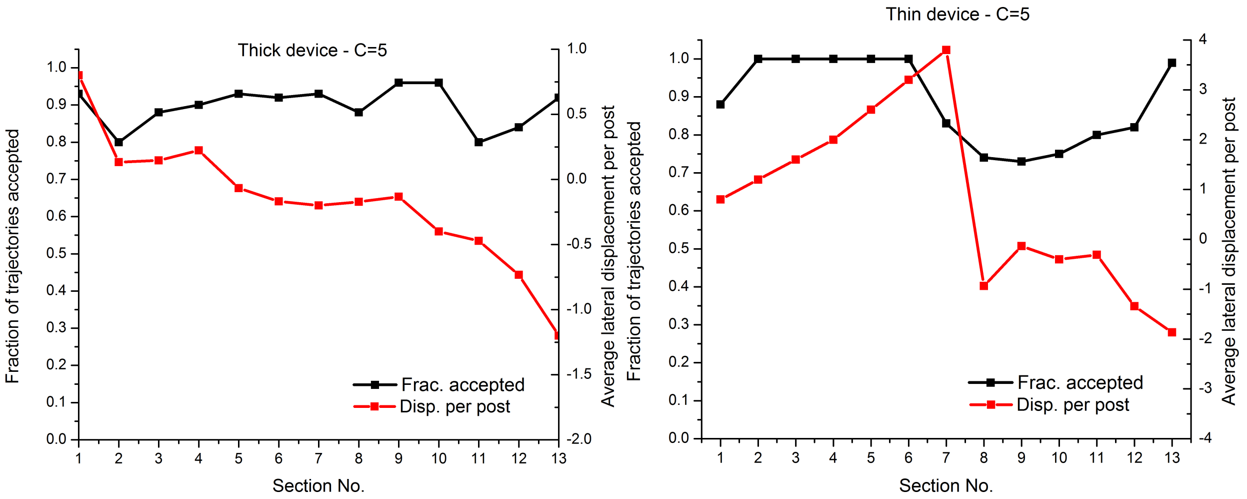

Supplementary Figure S3: (left) The fraction of trajectories accepted in each section of the thick device at $C = 5$. Note dips in the acceptance fraction which correspond to the transition from displacement to zig-zag and in later sections with relatively sensitive transit modes (see Supplementary Figure S4). **(right)** The fraction of trajectories accepted in each section of the thin device at $C = 5$. Note dip in the acceptance fraction corresponding to the transition from displacement to zigzag in sections 7-11 of the device. Note that the acceptance fractions for sections 2-5 are omitted as displacement-mode transit was evident from videos.

patterns above correspond to just portions of a full transit mode in this section. Currently, we cannot rule out one or another possibility. In addition, Supplementary Figure S5 illustrates a rejected trajectory, where a blockage between pillars has been detected. This example corresponds to a displacement mode and the sudden jump in the trajectory is due to the blockage of a single inter-pillar space identified visually from the video.

2 Numerical methods

2.1 Fluid simulation - mesoscale hydrodynamics

To represent the suspending fluid, we employ a mesoscale hydrodynamic simulation approach which is a variation of the smoothed dissipative particle dynamics (SDPD) method [9], adapted to conserve angular momentum [10]. SDPD is a particle-based fluid-dynamics method [9] well suited to mesoscopic length scales. It improves upon the popular dissipative particle dynamic (DPD) method [11, 12], by incorporating the force scheme used in smoothed particle hydrodynamics [13, 14], which has been derived directly through the discretization of the Navier-Stokes (NS) equations. A caveat of the original SDPD method [9] is its violation of conservation of angular momentum caused by force components which act perpendicular to the inter-particle axis. Recent multi-particle collision dynamics simulations [15] and SDPD simulations [10] have demonstrated that conservation of angular momentum is necessary in order to properly describe the dynamics of two different fluid phases (e.g., extra- and intra-cellular fluids separated by a RBC membrane) in flow. Consequently, the original SDPD formulation has been extended to obtain a new SDPD+a method, which satisfies angular momentum conservation [10]. This method is necessary for accurate simulation of RBC dynamics in the DLD device.

A SDPD+a system contains N particles with mass m_i, moment of inertia I_i, position $\mathbf{r}_i$, translational velocity $\mathbf{v}_i$, and rotational velocity (or spin) $\boldsymbol{\omega}_i$. Discretization of the NS equation with spin [10] provides a set of pairwise forces

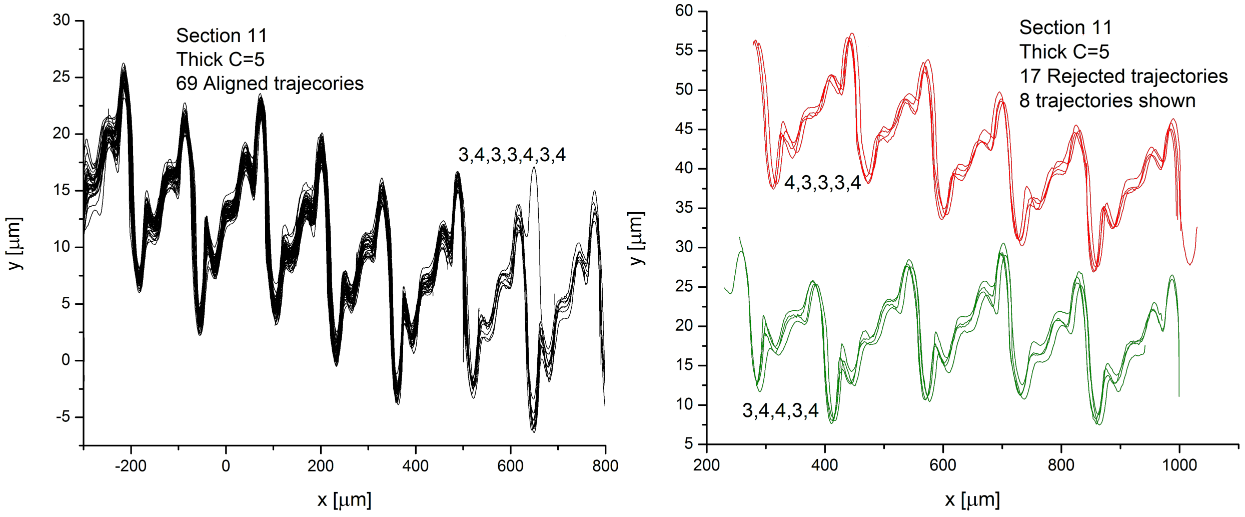

Supplementary Figure S4: (left) Example of the most prevalent trajectory in section 11 of the thick device at $C = 5$. 69 trajectories were aligned, showing the displacement-zigzag pattern {5,4,5,4,4,5,4}. **(right)** Illustration of the two minor populations of trajectories in section 11 of the thick device at $C = 5$. 17 rejected trajectories were aligned and found to either undergo the displacement-zigzag pattern {5,4,4,4,5} or {5,4,5,5,4}.

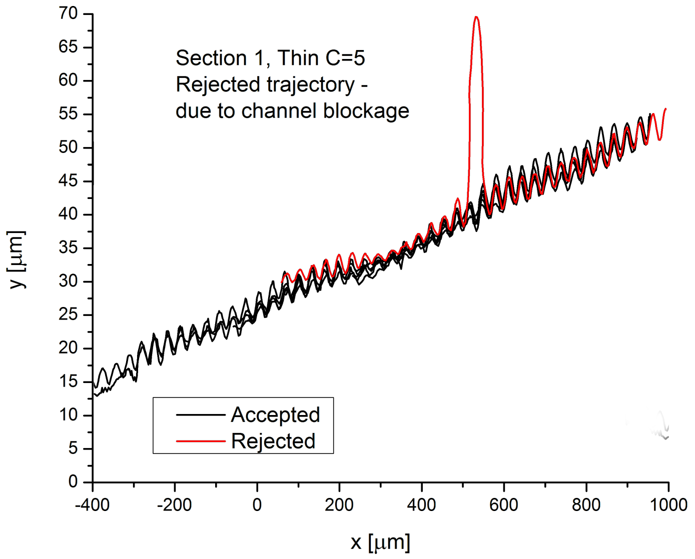

Supplementary Figure S5: A rejected trajectory due to a possible blockage between pillars.

Section	Total # of traj.	Rejected	Accepted	Accepted fraction	Average # of posts
1	27	2	25	0.93	46
2	25	5	20	0.8	67
3	17	2	15	0.88	37
4	10	1	9	0.9	30
5	15	1	14	0.93	27
6	12	1	11	0.92	26
7	28	2	26	0.93	25
8	25	3	22	0.88	32.2
9	23	1	22	0.96	28
10	50	2	48	0.96	22
11	86	17	69	0.80	22
12	134	21	113	0.84	19
13	97	8	89	0.92	14

Supplementary Table S2: The total number of RBC trajectories recorded in each section of the thick device at $C = 5$ and the number of excluded and accepted trajectories. The average number of posts describes the average length of all accepted trajectories.

which include conservative (C), dissipative (D), rotational (R), and stochastic (S) terms as follows

$$\begin{aligned}
\mathbf{F}_{ij}^C &= \left(\frac{p_i}{\rho_i^2} + \frac{p_j}{\rho_j^2}\right) w_{ij}\mathbf{r}_{ij}, \\
\mathbf{F}_{ij}^D &= -\gamma_{ij}\left(\mathbf{v}_{ij} + \frac{(\mathbf{v}_{ij}\cdot\hat{\mathbf{r}}_{ij})\,\hat{\mathbf{r}}_{ij}}{3}\right) + \frac{2\gamma_{ij}}{3}\left(\mathbf{v}_{ij}\cdot\hat{\mathbf{r}}_{ij}\right)\hat{\mathbf{r}}_{ij}, \\
\mathbf{F}_{ij}^R &= -\gamma_{ij}\frac{\mathbf{r}_{ij}}{2}\times\left(\boldsymbol{\omega}_i + \boldsymbol{\omega}_j\right), \\
\mathbf{F}_{ij}^S &= \sigma_{ij}\left(d\overline{\mathbf{W}}_{ij}^s + \frac{1}{3}tr\left[d\mathbf{W}_{ij}\right]\mathbb{1}\right)\cdot\frac{\hat{\mathbf{r}}_{ij}}{dt},
\end{aligned} \tag{2}$$

where p_i and p_j are local particle pressures given by the equation of state $p = p_0\,(\rho/\rho_0)^\alpha - b$, with p_0, ρ_0, α, and b being selected model parameters [10, 14]. Particle density ρ is calculated locally as $\rho_i = \sum_j W_L\,(r_{ij})$, where $W_L(r) = \frac{105}{16\pi r_c^3}\left(1 + 3\frac{r}{r_c}\right)\left(1 - \frac{r}{r_c}\right)^3$ is the Lucy function [13] and r_c is the cut-off radius. The weight function $w(r)$ is calculated by $\nabla W_L(r) = -\mathbf{r}w(r)$, such that $w_{ij} = w(r_{ij})$. The coefficients γ_{ij} and σ_{ij} determine the strength of the dissipative and random forces, where the friction coefficient γ_{ij} is defined as

$$\gamma_{ij} = \frac{20\eta_0}{7}\frac{w_{ij}}{\rho_i\rho_j}, \tag{3}$$

with η_0 being the desired dynamic viscosity. The random force coefficient is $\sigma_{ij} = 2\sqrt{k_B T\gamma_{ij}}$, while $tr[d\mathbf{W}_{ij}]$ and $d\overline{\mathbf{W}}_{ij}^s$ refer to the trace of a random matrix of independent Wiener increments $d\mathbf{W}_{ij}$ and its traceless symmetric part, respectively. For a formal derivation of these equations, the reader is referred to Ref. [10].

Newton's second law of motion governs the time evolution of the i-th particle's position, translational velocity, and rotational velocity as follows

$$\dot{\mathbf{r}}_i = \mathbf{v}_i, \quad \dot{\mathbf{v}}_i = \frac{1}{m_i}\sum_j \mathbf{F}_{ij}, \quad \dot{\boldsymbol{\omega}}_i = \frac{1}{I_i}\sum_j \mathbf{N}_{ij}, \tag{4}$$

where $\mathbf{N}_{ij} = \frac{1}{2}\mathbf{r}_{ij}\times\mathbf{F}_{ij}$ is the torque exerted on particle i by particle j. The equations of motion are integrated using the velocity-Verlet algorithm [16].

Section	Total # of traj.	Rejected	Accepted	Accepted fraction	Average # of posts
1	8	1	7	0.88	28
2	-	-	-	-	-
3	-	-	-	-	-
4	-	-	-	-	-
5	-	-	-	-	-
6	15	0	15	1.0	23
7	23	4	19	0.83	29
8	39	10	29	0.74	16
9	26	7	19	0.73	15
10	20	5	15	0.75	15
11	40	8	32	0.8	15
12	17	3	14	0.82	16
13	71	1	70	0.99	11

Supplementary Table S3: The total number of RBC trajectories recorded in each section of the thin device at $C = 5$ and the number of excluded and accepted trajectories. The average number of posts corresponds to the average length of all accepted trajectories. Note that values for sections 2-5 are omitted as displacement-mode transit was evident from videos and there was no need for alignment.

2.2 Red blood cell model - triangulated surfaces

The RBC membrane is modeled as a triangulated network of springs [17–21], whose vertices are coupled to the fluid via frictional forces. A total of N_v particles constitute the mesh vertices and $N_s = 3(N_v - 2)$ springs follow the edges of the mesh, reproducing the elasticity of the membrane (U_{sp}). A total of $N_t = 2N_v - 4$ triangles make up the entire membrane surface and incident triangles have an associated potential energy (U_{bend}) given by the angle between them, associated with membrane bending rigidity. Furthermore, local and global area constraints (U_{area}) are enforced along with a global volume constraint (U_{vol}). Formally, the total energy of a RBC is given as

$$U_{tot} = U_{sp} + U_{bend} + U_{area} + U_{vol}. \tag{5}$$

The total contribution of springs is given by

$$U_{sp} = \sum_{j\in 1\ldots N_s} \left[\frac{k_B T l_m \left(3x_j^2 - 2x_j^3\right)}{4\zeta\left(1 - x_j\right)} + \frac{k_p}{l_j} \right], \tag{6}$$

where l_j is the length of spring j, l_m is the maximum permitted spring extension, $x_j = l_j/l_m$ is the fractional extension towards maximum length, ζ is the persistence length, and k_p is the spring constant. The equilibrium length of each spring l_{0_j} is set in accordance with an initial triangulated mesh of a stress-free biconcave RBC shape [20, 21].

The membrane bending rigidity in absence of spontaneous curvature is described by a bending energy

$$U_{bend} = \sum_{j\in 1\ldots N_s} k_b \left[1 - \cos\theta_j\right], \tag{7}$$

where k_b is the bending constant and θ_j is the instantaneous angle between the two triangles incident on edge j.

Finally, the area and volume constraints are accounted for by two potentials:

$$U_{area} = k_a \frac{(A - A_r)^2}{2A_r} + \sum_{j\in 1\ldots N_t} k_d \frac{\left(A_j - A_j^0\right)^2}{2A_j^0}, \qquad U_{vol} = k_v \frac{(V - V_r)^2}{2V_r}, \tag{8}$$

where k_a, k_d, and k_v are the global area, local area, and volume constraint coefficients, respectively. A is the instantaneous surface area of the membrane, A_j is the instantaneous area of the j-th triangle in the network, and V is the

Section	Total # of traj.	Rejected	Accepted	Accepted fraction	Average # of posts
1	14	0	14	1.0	31
3	12	2	10	0.83	37
4	29	5	24	0.83	25
5	13	2	11	0.85	41
6	13	27	0	1.0	27
7	37	1	36	0.97	37
8	31	7	25	0.78	23
9	96	0	96	1.0	28
11	38	6	32	0.84	21
13	97	8	89	0.92	31

Supplementary Table S4: The total number of RBC trajectories recorded in each section of the thick device at $C = 1$ and the number of excluded and accepted trajectories. The average number of posts describes the average length of all accepted trajectories.

Section	Total # of traj.	Rejected	Accepted	Accepted fraction	Average # of posts
1	13	1	12	0.92	38
2	16	2	14	0.88	43
3	12	1	11	0.92	31
4	41	6	35	0.85	39
5	17	4	13	0.76	48
9	45	4	41	0.91	22
11	5	1	4	0.8	21

Supplementary Table S5: The total number of RBC trajectories recorded in each section of the thick device at $C = 0.25$ and the number of excluded and accepted trajectories. The average number of posts describes the average length of all accepted trajectories.

instantaneous RBC volume. The desired total surface area A_r, individual triangle area A_j^0, and interior volume V_r are set in accordance with the initial triangulation [20,21].

We relate the RBC model's variables to physical macroscopic properties of the RBC membrane by linear analysis for a regular hexagonal network [20,21]. The membrane shear modulus is related to the spring variables by

$$\mu_0 = \frac{\sqrt{3}k_BT}{4\zeta l_m x_0}\left(\frac{x_0}{2\left(1-x_0\right)^3} - \frac{1}{4\left(1-x_0\right)^2} + \frac{1}{4}\right) + \frac{3\sqrt{3}k_p}{4l_0^3}, \quad (9)$$

with $x_0 = l_0/l_m$. The area-compression K and Young's Y moduli are found as $K = 2\mu_0 + k_a + k_d$ and $Y = \frac{4K\mu_0}{K+\mu_0}$. The Helfrich model is employed to describe the bending coefficient k_b in terms of macroscopic bending rigidity κ [22], yielding $k_b = 2\kappa/\sqrt{3}$. The value of x_0 is set to 2.2 for all springs [21].

2.3 Simulation set-up and boundary conditions

The floor, ceiling, and pillar walls of the DLD device are modeled by a layer of frozen particles with a thickness of r_c which share the same equilibrium structure as the suspending fluid. This ensures that all particle interactions occurring in the locality of boundaries do not display artifacts (e.g., particle density variations) which could arise in the absence of wall particles due to an improper distribution of conservative forces. Furthermore, the wall particles are included in calculation of fluid particle densities ρ near the wall.

Section	Total # of traj.	Rejected	Accepted	Accepted fraction	Average # of posts
1	14	2	12	0.86	27
3	6	0	6	1.0	45
5	13	0	13	1.0	28
7	11	0	11	1.0	12
9	23	2	21	0.91	9

Supplementary Table S6: The total number of RBC trajectories recorded in each section of the thin device at $C = 2$ and the number of excluded and accepted trajectories. The average number of posts corresponds to the average length of all accepted trajectories.

In order to prevent particles from penetrating the walls, RBC vertices and fluid particles are subject to bounce-back reflections at walls. Bounce-back reflections are preferred over specular reflections as they achieve a better approximation of no-slip boundary conditions (BCs) at the wall. Finally, to fully guarantee no-slip BCs at the walls, an adaptive tangential force is applied to fluid particles within a distance r_c from the walls [23].

To prevent mixing between intra- and extra-cellular fluids, bounce-back reflections for the solvent particles are also introduced at a RBC membrane. Furthermore, the RBC is coupled to fluid flow via viscous friction between the N_v mesh vertices and local fluid particles. The dissipative and random force components of the DPD method are used to achieve these interactions [20]. No-slip BCs at membrane vertices are enforced by careful selection of the friction parameter γ in the dissipative force $\mathbf{F}^D$. A fluid sheared over the effective surface of a membrane vertex exerts a friction force on the membrane given as $\mathbf{F}_v = \int_{V_h} ng(r)\mathbf{F}^D dV$, where n is the fluid number density, $g(r)$ is the radial distribution function of fluid particles about the membrane vertices, and V_h is the hemisphere volume of fluid situated above the vertex. Equating this integral to the total force required by a continuum hydrodynamical description leads to an expression for the calculation of γ [20].

The bumper array is simulated using a 3D domain enclosing a single-column obstacle with its axis in the z direction, perpendicular to the roof and floor of the domain box, as shown in Supplementary Figure. S6. Efficient representation of an infinite bumper array environment is achieved with periodic BCs in the x and y directions and a shift in the y direction for each boundary-crossing event in the x direction. The assumption of an infinite obstacle array neglects any effects induced by the walls at either edge of the device. The missing wall effects are revealed by solving the NS equation for a bumper array in 2D with explicit wall-edge boundaries. We find that the edge walls enforce a zero net flow in the y direction. This condition is recreated in the 3D infinite array using an adaptive force applied to solvent particles such that the net flow in the y direction vanishes.

Simulations are performed for every section of the device, altering the lateral shift $\Delta\lambda$ induced at the periodic boundary to correspond with a specific section of the experimental device, as shown in Supplementary Table S1. This gives us a total of 13 trajectories for a single RBC transit through the 13 sections of each thin ($H = 4\,\mu\text{m}$) and thick ($H = 11\,\mu\text{m}$) device.

Establishing standard length and energy scales allows us to relate most simulation parameters to physical RBC properties. We use the RBC membrane area to define an effective RBC diameter $D_r = \sqrt{A_r/\pi}$ and an average bond length for a given number of vertices $l_0^2 = \frac{4\sqrt{3}A_r}{3N_t}$. The experimental value for an average healthy RBC's surface area $A_r = 133.5 \times 10^{-12}\,\text{m}^2$ [24] suggests a $D_r = 6.5\,\mu\text{m}$. For time scaling, we define a characteristic RBC relaxation time $\tau = \eta_o D_r^3/\kappa_r$, where η_o is the suspending fluid viscosity and κ_r is the RBC membrane bending rigidity. The parameters used for the RBC model are summarized within Supplementary Table S6 in units of D_r and the thermal energy k_BT; along side we present the corresponding average values for a healthy RBC in physical units.

The SDPD+a fluid parameters are given in Supplementary Table S7. The average distance between RBC vertices l_0 is chosen as the length scale, which has a value of $l_0 = 0.4$ in simulations. The exponent α in the equation of state takes the value $\alpha = 7$ and the SDPD equilibrium density $\rho_0 = n$, where $n = 9$ is the fluid's number density. The speed of sound for the equation of state is given as $c^2 = p_0\alpha/\rho_0$ and the Mach number has been kept below 0.1 in all simulations, ensuring a good approximation for incompressible fluid flow.

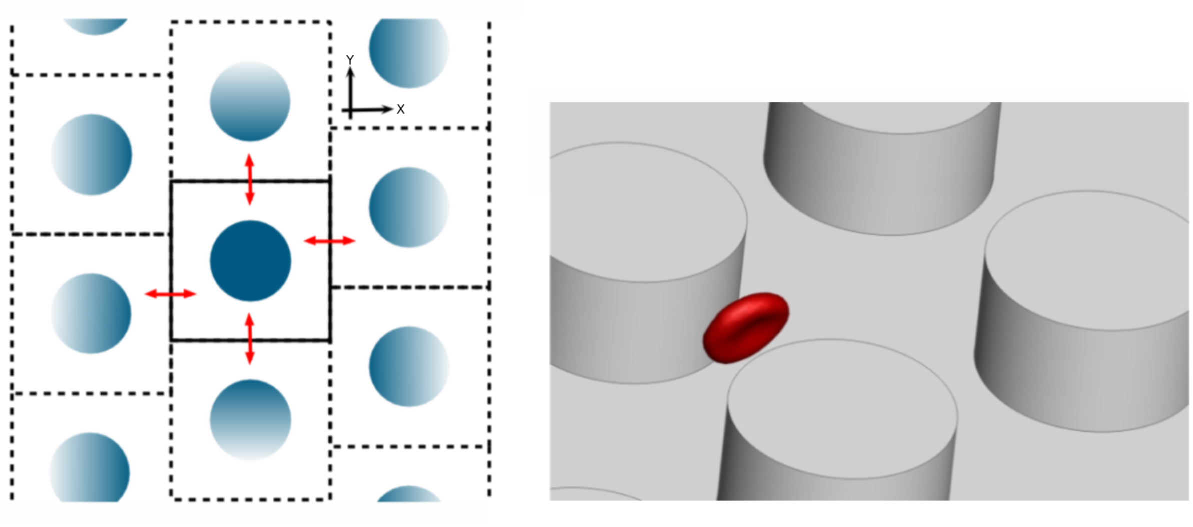

Supplementary Figure S6: **(left)** Top-down schematic of the simulated domain. The solid lines enclose a square of the simulation domain and the dotted lines denote the periodic BCs with a shift in the y direction. **(right)** A snapshot of a simulated RBC in the obstacle array.

3 Supplementary videos

In order to best demonstrate the differences in RBC dynamics for cells with viscosity contrasts $C = 5$ and $C = 1$, we present several videos of simulated and experimental RBC transit in sections 4 and 11 of the *thick* device.

3.1 Viscosity contrast $C = 5$

The three videos "SIM_Sec4Cont5.avi", "EXP_Sec4Cont5.avi", and "SIM_Sec11Cont5.avi" show the behavior of simulated and experimental RBCs traveling through sections 4 and 11 of the *thick* device in a suspending medium which is five times less viscous than the intracellular fluid. Notice that row-swapping events occur; the RBCs are in a zig-zag mode. The RBCs display tumbling dynamics, which is especially prevalent before and after row-swapping events.

3.2 Viscosity contrast $C = 1$

The other two videos "SIM_Sec4Cont1.avi" and "EXP_Sec4Cont1.avi" also show the simulated and experimental RBCs traveling through section 4 of the thick device. However, the inner and outer fluids have the same viscosity and as a result, we see RBCs traveling in displacement modes without zig-zagging. The RBCs tumble to a lesser degree and there are clear local deformations as the cell membrane rearranges itself in a tank treading motion.

References

[1] J. P. Beech, S. H. Holm, K. Adolfsson, and J. O. Tegenfeldt. Sorting cells by size, shape and deformability. *Lab Chip*, 12:1048–1051, 2012.

[2] S. H. Holm, J. P. Beech, M. P. Barrett, and J. O. Tegenfeldt. Separation of parasites from human blood using deterministic lateral displacement. *Lab Chip*, 11:1326–1332, 2011.

[3] B. R. Long, M. Heller, J. P. Beech, H. Linke, H. Bruus, and J. O. Tegenfeldt. Multidirectional sorting modes in deterministic lateral displacement devices. *Phys. Rev. E*, 78:046304, 2008.

[4] J. Frechette and G. Drazer. Directional locking and deterministic separation in periodic arrays. *J. Fluid Mech.*, 627:379–401, 2009.

RBC parameters	Scaled units	Physical units
N_v	1000	
A_r		$133.5 \times 10^{-12}\,\mathrm{m}^2$
D_r	$\sqrt{A_r/\pi}$	$6.5 \times 10^{-6}\,\mathrm{m}$
l_0	$0.061\,D_r$	$3.91 \times 10^{-7}\,\mathrm{m}$
V_r	$0.34\,D_r^3$	$93.1 \times 10^{-18}\,\mathrm{m}^3$
T		$310\,\mathrm{K}$
Y_r	$1.82 \times 10^5 \frac{k_B T}{D_r^2}$	$18.9 \times 10^{-6}\,\mathrm{N/m}$
κ_r	$70\,k_B T$	$3 \times 10^{-19}\,\mathrm{J}$
k_d	$4.2 \times 10^4 \frac{k_B T}{D_r^2}$	$4.3 \times 10^{-6}\,\mathrm{N/m}$
k_a	$2.1 \times 10^6 \frac{k_B T}{D_r^2}$	$2.1 \times 10^{-4}\,\mathrm{N/m}$
k_v	$1.4 \times 10^7 \frac{k_B T}{D_r^3}$	$220\,\mathrm{N/m}^2$

Supplementary Table S7: Parameters defining the model values used for the RBC properties and their physical equivalents. N_v is the number of membrane vertices, A_r is the RBC membrane area, l_0 is the average bond length, V_r is the RBC volume, T is the temperature, Y_r is the membrane Young's modulus, κ_r is the membrane bending rigidity, and k_d, k_a, and k_v are the local area, global area, and volume constraint coefficients, respectively. In all simulations, we have chosen $A_r = 133.5$ and $k_B T = 0.4$, which implies that $D_r = 6.5$ and $l_0 = 0.4$.

Fluid parameters	Scaled units	Physical units
p_0	$16 \frac{k_B T}{l_0^3}$	$1.07\,\mathrm{Pa}$
b	$12.8 \frac{k_B T}{l_0^3}$	$0.86\,\mathrm{Pa}$
η_o	$25.3 \frac{\sqrt{m k_B T}}{l_0^2}$	$1.2 \times 10^{-3}\,\mathrm{Pa \cdot s}$
η_i	$25.3, 126.6 \frac{\sqrt{m k_B T}}{l_0^2}$	$6 \times 10^{-3}\,\mathrm{Pa \cdot s}$
$k_B T$		$4.282 \times 10^{-21}\,\mathrm{J}$

Supplementary Table S8: Parameters defining the model values used for the fluid properties and their physical equivalents. Mass and length for SDPD+a fluid are measured in units of the fluid particle mass m and the membrane bond length l_0. p_0 and b are parameters for the pressure equation, and η_o and η_i are the extra- and intra-cellular fluid dynamic viscosities, respectively. In all simulations, we have set $m = 1$, $l_0 = 0.4$, and the thermal energy $k_B T = 0.4$.

[5] L. R. Huang, E. C. Cox, R. H. Austin, and J. C. Sturm. Continuous particle separation through deterministic lateral displacement. *Science*, 304:987–990, 2004.

[6] D. W. Inglis, J. A. Davis, R. H. Austin, and J. C. Sturm. Critical particle size for fractionation by deterministic lateral displacement. *Lab Chip*, 6:655–658, 2006.

[7] C. A. Schneider, W. S. Rasband, and K. W. Eliceiri. Real-time deformability cytometry: on-the-fly cell mechanical phenotyping. *Nat. Methods*, 9:671–675, 2012.

[8] D. P. Huttenlocher, G. A. Klanderman, and W. J. Rucklidge. Comparing images using the Hausdorff distance. *IEEE T. Pattern Anal.*, 15:850–863, 1993.

[9] P. Español and M. Revenga. Smoothed dissipative particle dynamics. *Phys. Rev. E*, 67:026705, 2003.

[10] K. Müller, D. A. Fedosov, and G. Gompper. Smoothed dissipative particle dynamics with angular momentum conservation. *J. Comp. Phys.*, 281:301–315, 2015.

[11] P. J. Hoogerbrugge and J. M. V. A. Koelman. Simulating microscopic hydrodynamic phenomena with dissipative particle dynamics. *Europhys. Lett.*, 19:155–160, 1992.

[12] P. Español and P. Warren. Statistical mechanics of dissipative particle dynamics. *Europhys. Lett.*, 30:191–196, 1995.

[13] L. B. Lucy. A numerical approach to the testing the fission hypothesis. *Astronom. J.*, 82:1013–1024, 1977.

[14] J. J. Monaghan. Smoothed particle hydrodynamics. *Annu. Rev. Astron. Astrophys.*, 30:543–574, 1992.

[15] I. O. Götze, H. Noguchi, and G. Gompper. Relevance of angular momentum conservation in mesoscale hydrodynamics simulations. *Phys. Rev. E*, 76:046705, 2007.

[16] M. P. Allen and D. J. Tildesley. *Computer simulation of liquids.* Clarendon Press, New York, 1991.

[17] G. Gompper and D. M. Kroll. Network models of fluid, hexatic and polymerized membranes. *J. Phys.: Condens. Matter*, 9:8795–8834, 1997.

[18] D. E. Discher, D. H. Boal, and S. K. Boey. Simulations of the erythrocyte cytoskeleton at large deformation. II. Micropipette aspiration. *Biophys. J.*, 75:1584–1597, 1998.

[19] H. Noguchi and G. Gompper. Shape transitions of fluid vesicles and red blood cells in capillary flows. *Proc. Natl. Acad. Sci. USA*, 102:14159–14164, 2005.

[20] D. A. Fedosov, B. Caswell, and G. E. Karniadakis. A multiscale red blood cell model with accurate mechanics, rheology, and dynamics. *Biophys. J.*, 98:2215–2225, 2010.

[21] D. A. Fedosov, B. Caswell, and G. E. Karniadakis. Systematic coarse-graining of spectrin-level red blood cell models. *Comput. Meth. Appl. Mech. Eng.*, 199:1937–1948, 2010.

[22] W. Helfrich. Elastic properties of lipid bilayers: theory and possible experiments. *Z. Naturforschung C*, 28:693–703, 1973.

[23] D. A. Fedosov and G. E. Karniadakis. Triple-decker: Interfacing atomistic-mesoscopic-continuum flow regimes. *J. Comp. Phys.*, 228:1157–1171, 2009.

[24] E. A. Evans and R. Skalak. *Mechanics and thermodynamics of biomembranes.* CRC Press, Inc., Boca Raton, Florida, 1980.
